# Metformin and berberine, two versatile drugs in treatment of common metabolic diseases

**DOI:** 10.18632/oncotarget.20807

**Published:** 2017-09-11

**Authors:** Haoran Wang, Chen Zhu, Ying Ying, Lingyu Luo, Deqiang Huang, Zhijun Luo

**Affiliations:** ^1^ Department of Gastroenterology, Research Institute of Digestive Diseases, The First Hospital of Nanchang University, Nanchang, China; ^2^ Jiangxi Provincial Key Laboratory of Tumour Pathogenesis and Molecular Pathology, Department of Pathophysiology, School of Basic Medical Sciences, Nanchang University, Nanchang, China; ^3^ Department of Biochemistry, Boston University School of Medicine, Boston, MA, USA

**Keywords:** metformin, berberine, metabolic diseases, tumour

## Abstract

Metformin has been used as a glucose lowering drug for several centuries and is now a first-line drug for type 2 diabetes mellitus (T2DM). Since the discovery that it activates AMP-activated protein kinase (AMPK) and reduces risk of cancer, metformin has drawn great attentions. Another drug, berberine, extracted from *berberis vulgaris L.* (root), was an ancient herbal medicine in treating diarrhea. Ongoing experimental and clinical studies have illuminated great potential of berberine in regulation of glucose and lipid homeostasis, cancer growth and inflammation. Furthermore, the lipid lowering effect of berberine is comparable to those conventional lipid drugs but with low toxicity. Therefore, it is right time to transform beneficial effects of berberine into therapeutic practice. Metformin and berberine share many features in actions despite different structure and both could be excellent drugs in treating T2DM, obesity, cardiac diseases, tumour, as well as inflammation. Since these disorders are often connected and comprise common pathogenic factors that could be targeted by the two drugs, understanding their actions can give us rationale for expansion of their clinical uses.

## INTRODUCTION

**Figure 1:**
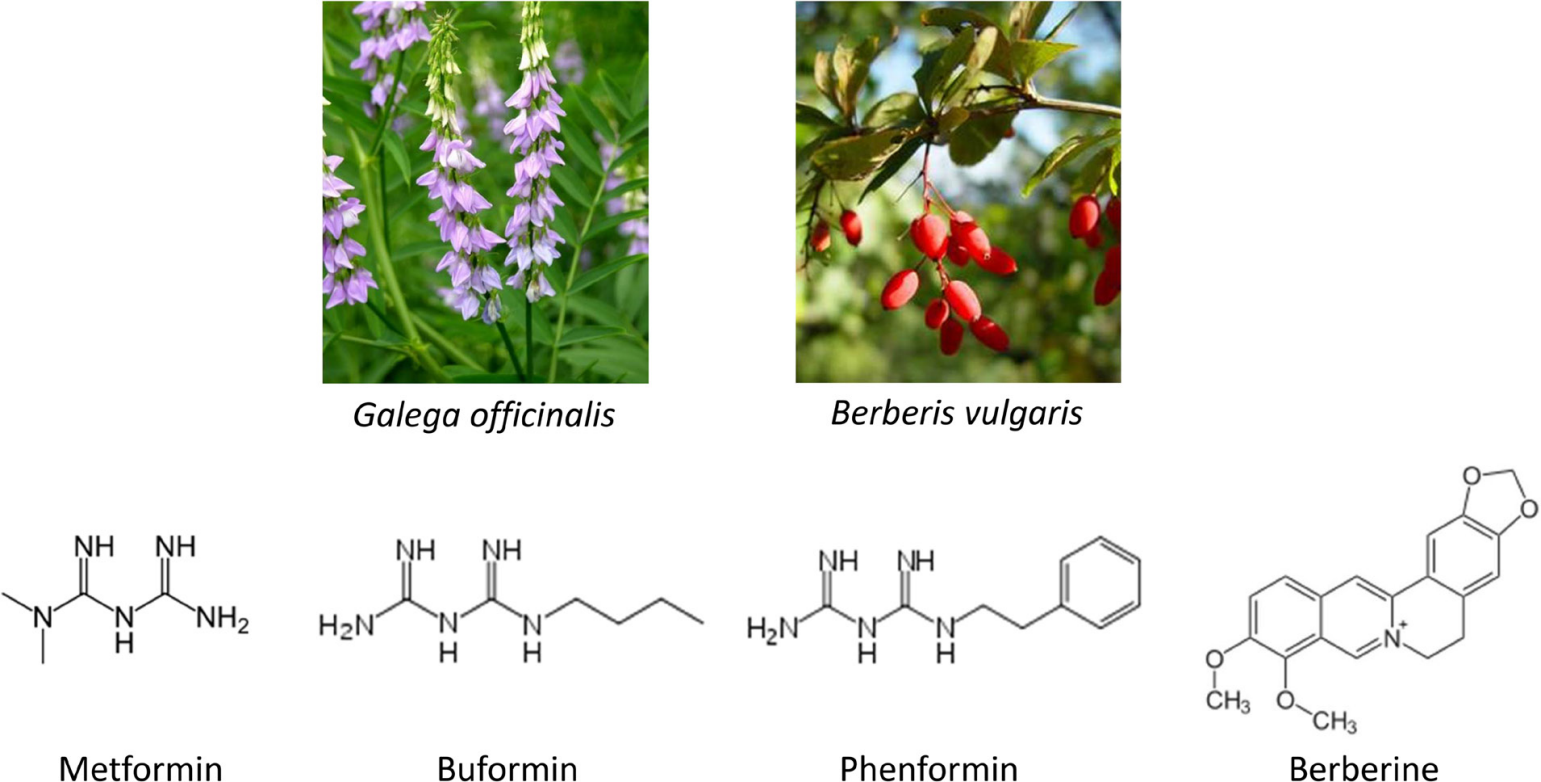
Structure of biguanides and berberine

The discovery of metformin dates back to 17th century. *Galega officinalis L.,* also known as the French lilac, was used as a herbal remedy to relieve the intense urination caused by the diabetes mellitus in medieval times [1]. The guanides are rich in French lilac and essential compounds in lowering blood glucose, which led to development of three biguanides, metformin, phenformn, and buformin (Figure 1). Among them metformin was found to be the most useful drug because of its low toxicity. It was first synthesized in 1922, but approved for treatment of diabetes in Europe until 1950s and by FDA in USA in 1994 [2]. Phenformin and buformin were abandoned in 1970s for intolerable side effects such as high frequency of lactic acidosis and increased mortality. The United Kingdom Prospective Diabetes Study (UKPDS) has revealed that metformin is the only oral anti-hyperglycemic agent that reduces macrovascular complications in patients with T2DM [3]. Further clinical investigations and practice have recommended it as a first line drug for T2DM. Moreover, it has been expanded to treatment of other diseases such as obesity and tumour.

Berberine was used in Chinese and Ayurvedic medicine around 3000 BC (Figure 1). The plant barberry (*berberis vulgaris*) was a herbal medicine in treating diarrhea [4] and dysentery [5]. Its constituents were then extracted, among which berberine was the most active alkaloid [6]. Berberine belongs to the structural class of protoberberines and is also present in the barks of other plant species including *Hydrastis Canadensis L.*, *Coptis chinensis Franch*, *Arcangelisia flava (L.) Meer., B. aquifolium Pursh.* and *B. aristata D.C.* [7], although the extraction purity of berberine varies among plants. Up to date, berberine has been used as a non-prescription drug in clinics for diarrhea, dysentery, stomatitis [8] and hepatitis [9]. Numerous researches have been carried out to unravel its other pharmacological and therapeutic effects, especially on T2DM, lipid metabolism and tumour.

Nowadays, diabetes has become one of the most common public health issues due to increased prevalence, morbidity and mortality caused by complications [10]. Tremendous amount of efforts has been casted to identify effective agents with low toxicity. Although many studies have shown that berberine exerts hypoglycemic actions similarly to metformin, it has not been extensively used in treating diabetes. Furthermore, the potentials of both drugs in obesity and tumour management have attracted a lot of research interests. Hence, in this review, we will summarize recent progresses in studies on mechanisms of their actions and compare their utilities in treatment of several common and interconnected diseases such as diabetes, obesity, cardiovascular diseases, and tumours as well as inflammation.

## ANTI-DIABETIC ACTION

Diabetes mellitus is a chronic, progressive metabolic disorder. There are two categories, type 1 diabetes mellitus (T1DM) and type 2 diabetes mellitus (T2DM), of which T2DM comprises almost 90% cases [11]. While T1DM is an early onset autoimmune disease that leads to destruction of β-cells, T2DM exhibits insulin resistance in liver, muscle and adipose at early stages, and eventually β cell failure at late stages, which is characterized as non-insulin dependent diabetes mellitus (NIDDM) [12]. The progression of T2DM starts from insulin resistance attributable to genetic and environmental factors [13]. Defects in insulin action at first can be compensated by increased insulin secretion, resulting in hyperinsulinemia, so as to maintain blood glucose homeostasis. Until the secretory function of the β cells cannot compensate further declined insulin sensitivity, the overt T2DM develops. In liver, insulin resistance is manifested by excessive production of glucose (gluconeogenesis) in the fasting state and impaired glucose uptake after meal regardless the presence of insulin, while in muscle, insulin resistance exhibits decreased glucose uptake, all of which lead to postprandial hyperglycemia [12, 14–16]. Liver, muscle and β cells were initially referred as the triumvirate of T2DM and are the targets of conventional therapies [12]. Later studies have provided evidence that adipose, brain, pancreatic α-cells, intestinal cells, and kidney also play important roles in the progression of T2DM [16].

Metformin suppresses hepatic glucose production and stimulates glucose uptake in muscle and adipose, resulting in improvement of hyperglycemia and hyperlipidemia and alleviating non-alcoholic fat liver disease (NAFLD) [17–19]. In addition to enhancing insulin sensitivity in peripheral tissues, metformin can protect β-islets against lipotoxicity and glucotoxicity to restore insulin secretion [20, 21]. The first target of metformin identified is the 5′-AMP activated protein kinase (AMPK), although some effects are reported to be mediated through AMPK-independent mechanisms [22–24].

The effects of berberine on T2DM was first reported in 1986 [25, 26]. Yin et al compared the effects of berberine and metformin [27]. In a three months trial, 36 patients with T2DM were randomly assigned with berberine or metformin. It was found that hypoglycemic effect of berberine is comparable to that of metformin. The level of hemoglobin A1c (HbA1c), fasting and postprandial glucose decreased by 7.5%, 6.9% and 11.1% respectively at the end of the trial. Similar findings were reported in a clinical study of Zhang et al [28]. A meta-analysis of 21 clinical trials revealed that berberine has therapeutic effects on T2DM, hyperlipidemia and hypertension, comparable to other therapeutic regimes [29]. Studies have indicated that, similarly to metformin, berberine executes its functions by regulating a variety of effectors including AMPK, MAPK, PKC, PPARα, PPARγ [28, 30]. To be noteworthy, via activation of AMPK, berberine can stimulate glucose uptake in muscle, liver and adipose, and inhibit gluconeogenesis in liver by downregulation of gluconeogenic enzymes (phosphoenolpyruvate carboxyl kinase and glucose-6-phosphatase) [31].

## ANTI-OBESITY

The effect of metformin on body weight and NAFLD has been assessed in a number of studies with inconclusive results. A 10-year follow-up study on patients with T2MD and obesity revealed modest body weight loss in groups treated with metformin, as compared to placebo [32]. However, Seifarth et al showed reduction of body weight in obese individuals without T2DM, in which treatment with metformin for 6 month caused a mean weight loss of 5.8 ± 7.0 kg, while untreated control group gained 0.8±3.5kg on average [33]. Le and Lomba [34] summarized results of eight studies on the effect of metformin on body weight and NAFLD/NASH and showed no consistent results (with the 50% to 50% ratio significance and insignificance), suggesting that more factors should be considered in experimental designs.

Berberine has been shown to be a potential drug to treat obesity by downregulation of adipognesis and lipogenesis. Mice treated with berberine were found to contain shrunk adipocytes [35]. This anti-obese activity is consistent with the finding that berberine significantly decreased sizes and number of lipid droplets in 3T3-L1 adipocytes [36]. Berberine exerts its long-term body weight losing effect through enhancing AMPK-mediated ATGL expression, which increases the basal lipolysis state of triglycerides in adipocytes [36]. In addition, berberine displays an inhibitory effect on proliferation and differentiation of preadipocytes. PPARγ is a crucial transcription factor of adipogenesis and berberine inhibits adipocyte differentiation through PPARγ and C/EBPα [37, 38].

Berberine was reported to inhibit cholesterol and triglyceride synthesis in HepG2 cells, a human hepatoma cell line, and primary hepatocytes [39, 40]. The evidence for the inhibitory effect of berberine on NAFLD comes from rodent animal models that are induced by fat diet [41–46]. There is no direct evidence in humans showing the protective effect of berberine on NAFLD, but an indirect clinical investigation suggests that berberine supplement may suppress NAFLD, as it reduces alanine and aspartate transaminase levels in patients with T2DM [47].

## CARDIOVASCULAR PROTECTION AGAINST DAMAGE BY HYPERLIPIDEMIA

Cardiovascular disease (CVD) has become one of the most severe complications of T2DM, an important factor for mortality. Thus, for patients with T2DM, cardiovascular protection is extremely important. In 1998, the UKPDS demonstrated that monotherapy of metformin was correlated to decreased risk of CVD in overweight T2DM patients [48]. Studies have shown that metformin significantly reduces free fatty acid, triglyceride and soluble vascular cell adhesion molecule-1 (sVCAM-1) levels in body, which account for decreases in the risk of CVD [49]. In a 7-year follow-up study, Xu et al assessed the effects of metformin on metabolite profiles and LDL cholesterol (LDL-c) [50]. The results revealed a lower blood level of LDL-c in T2DM patients treated with metformin, compared to other groups. Hyperlipidemia, especially LDL-c, has been proven to be a risk factor of coronary heart diseases [51]. Therefore, metformin can reduce the risk of coronary heart diseases complicated with T2DM.

The documentation of the beneficial effects of berberine on CVD dates back to the 1980s [52]. Intravenous infusion of berberine in 12 patients with heart failure refractory to digitalis and diuretics led to an acute decrease in peripheral resistance and increase in cardiac index. This finding has inspired researchers’ interests. Berberine was first reported to lower LDL-c, a risk factor for CVD, by upregulating LDLR gene expression and stability of LDLR mRNA and therefore increasing LDLR-mediated liver clearance [53, 54]. Another study showed that in elderly hypercholesterolemic patients who were statin-intolerant, berberine could ameliorate hypercholesterolemia and plasma LDL-c levels [55].

With regard to the mechanism, it has been reported that berberine modulates LDLR at a post-translational level [56, 57]. In HepG2 cells, berberine causes ubiquitination and degradation of hepatocyte nuclear factor 1α (HNF1α), which is a critical transcription activator of proprotein convertase subtilisin/kexin type 9 (PCSK9), a nature inhibitor of LDLR. PCSK9 binds to the extracellular domain of LDLR, causing its degradation. Thus, LDLR is activated as a result of reduced expression of PCSK9. In addition, berberine lowers blood cholesterol levels through inhibiting intestinal absorption, cholesterol uptake and secretion in enterocyte [58]. The amphipathic property of berberine interferes with cholesterol micellarization in the intestinal lumen, thus decreasing absorption. Similar to the effect on micelles, this physical-chemical property may interact with enterocyte membrane, decreasing permeability of cholesterol micelles and in turn the cholesterol uptake. Following micelles entering the enterocytes, re-esterification step is also interfered because berberine downregulates the gene expression of Acetyl-CoA acetyltransferase 2, leading to decreased secretion of cholesterol from enterocytes into the lymphatics. These results are in accordance with the observations in berberine-fed rats that the levels of total plasma cholesterol, LDL-c and dietary cholesterol absorption rate decreased by 31%, 36% and 45%, respectively [58]. Above all, the lipid lowering effects of berberine sound more attractive than those conventional lipid lowering drugs because of its low toxicity and its pairing with drugs like statins could improve the therapeutic efficacy and life quality of hyperlipidemic patients [29].

## ANTI-TUMOUR ACTIVITY

**Figure 2:**
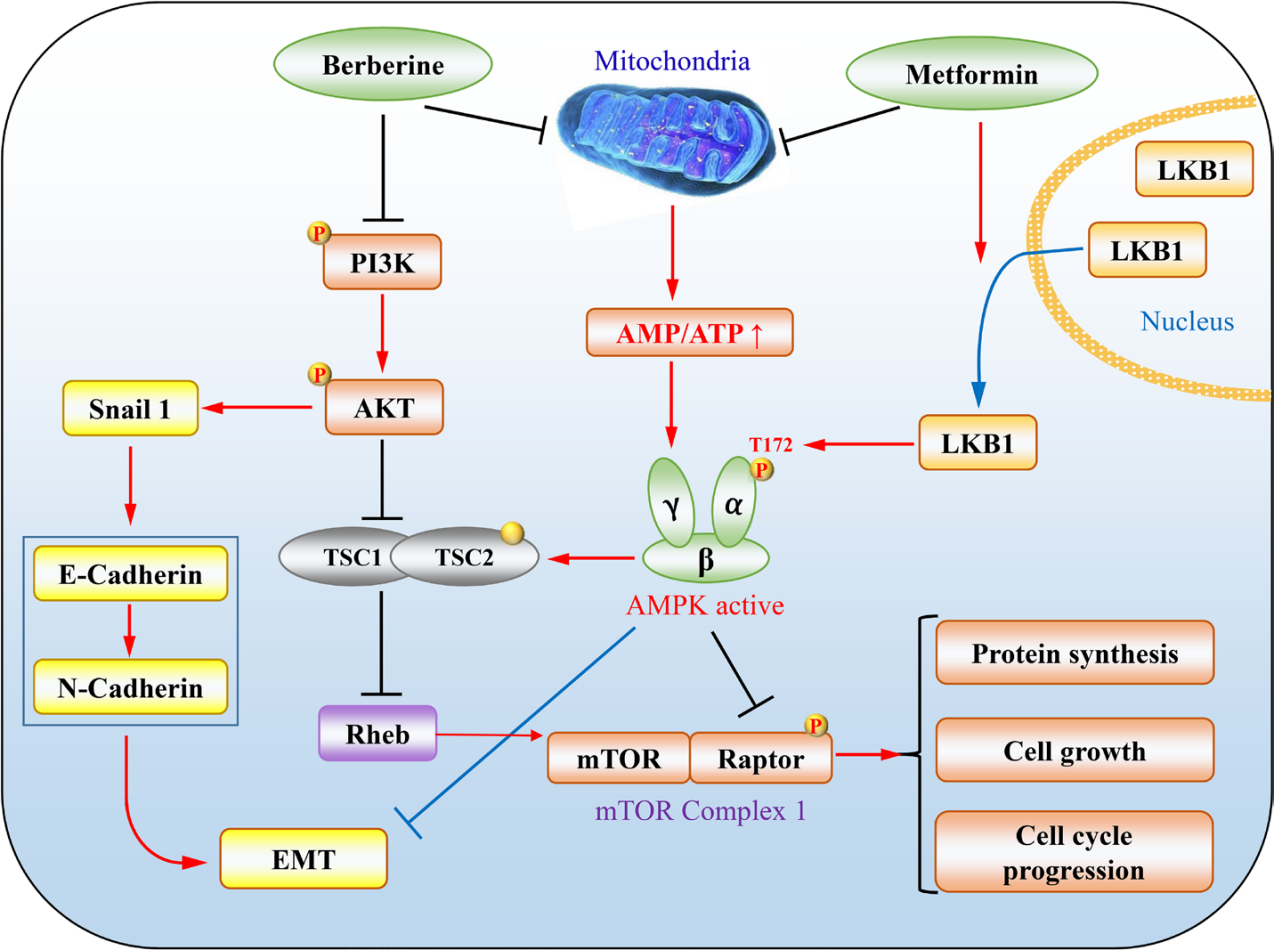
The representative mechanisms underlying tumour suppression by metformin and berberine (1) LKB1/AMPK/mTORC1 axis. PI3K/Akt inhibits TSC1/TSC2, leading to increases in Rheb-GTP and mTOR activation. Metformin increases levels of AMP and thus promotes activation of AMPK through LKB1 phosphorylation of T172, which by phosphorylation in turn inhibits raptor and activates the TSC1/TSC2 complex, an GTPase activating protein for Rheb, culminating in inhibition of mTORC1. (2) Both metformin and berberine can attenuate EMT. In addition, berberine activates AMPK to execute cellular functions by a mechanism similarly to metformin.

The effects of metformin on tumour have emerged as a hot topic during the last decade or so [24, 59, 60]. The first seminal report is the retrospective investigation on the incidence of tumour in T2DM patients receiving metformin, which shows 30% reduction of overall tumour onsets [61]. The T2DM patients with cancers such as lung cancer, pancreatic cancer and breast cancer showed better prognosis when metformin was used as a hypoglycemic drug [62–64]. A plethora of *in vitro* studies have demonstrated that metformin inhibit tumour cell growth via various mechanisms [24, 60]. Therein, AMPK plays an important role in mediating the tumour suppressing effect of metformin. First, the liver kinase B1 (LKB1), a dominant upstream kinase of AMPK that phosphorylates Thr-172 in the activation loop, is a tumour suppressor. Loss-of-function mutations of LKB1 have been found in many types of cancer, or its gene is hypermethylated and suppressed if not mutated [60, 65, 66]. Although LKB1 is not activated directly by metformin, it is required for maximal activation of AMPK. As such, the anti-proliferative action of metformin is compromised in tumour/cells lacking LKB1 (Figure 2) [60]. Moreover, it has been reported that metformin induces cytoplasmic translocation of LKB1, enhancing AMPK activation [67]. Second, the AMPK pathway targets many key tumour-promoting signalling pathways, one of which is the mammalian target of rapamycin (mTOR). mTOR is an important protein kinase which regulates protein translation and cell cycle progression. This kinase is bound with accessary proteins such as raptor to form mTOR complex 1 (mTORC1) [68]. GTP-bound Ras homolog enriched in brain (Rheb) is a necessary mTOR activator that is inhibited by tuberous sclerosis complex 1 (TSC1) and tuberous sclerosis complex 2 (TSC2). These two proteins form a GTPase activating protein (GAP) with specificity toward Rheb [69]. In tumour cells bearing activated Akt, TSC2 is inhibited by phosphorylation, leading to an increase in Rheb-GTP and mTOR activation. AMPK inhibits mTOR mainly via two mechanisms, phosphorylation and activation of TSC2, and phosphorylation and inhibition of Raptor. In addition, effector proteins of AMPK implicated in cell growth have increased to a long list [70]. Therefore, the activation of LKB1/AMPK pathway by metformin offers a meaningful strategy for tumour treatment.

Likewise, the antitumor activity of berberine has become increasingly interesting. Up till now, there are more than 600 publications in PubMed by entering “berberine and cancer”. Berberine has been shown to exert antitumor effects through multiple routes, for example, suppressing cell proliferation, metastasis and angiogenesis in numerous tumour types, including breast cancer, gastric cancer, melanoma, and hepatoma [71–76]. Studies of Yu et al have shown that berberine can reverse epithelial mesenchymal transition (EMT) and suppress invasion and migration of the mouse melanoma B16 cells by 50.5% and 67.53%, respectively [73]. Berberine decreases levels of phosphorylated PI3K (p-PI3K), p-AKT and RARα while increases RARβ, RARγ and the epithelial marker E-cadherin. Through regulation of RARα/β and the PI3K/AKT signalling pathway, berberine could be used as an adjuvant therapeutic agent to metastatic melanoma that cannot be cured only by surgery. Similar therapeutic potential is also proposed in triple negative basal-like breast cancers, most malignant breast cancer subtype [77–79]. Berberine can target vasodilator-stimulated phosphoprotein, which mediates cell migration and whose elevation level is correlated with poor pathological stage [71, 80]. In terms of possible role in inhibiting EMT and metastasis of cancer, berberine and metformin seem to have similar effects [81–83].

## ANTI-INFLAMMATORY EFFECT

Nowadays, it is accepted that dysregulation of immunity is involved in diabetes, obesity, cardiovascular diseases and cancer. Infiltration of immune cells such as macrophages and neutrophils to affected tissues, where cytokines are secreted plays important roles in the pathogenesis of the diseases. The anti-inflammatory effect of berberine has been acknowledged for long history. Current investigations have revealed that berberine exerts the anti-inflammatory activities in the intestinal lumen by regulating their transcription and therefore ameliorating pro-inflammatory cytokine-induced intestinal epithelial damage, which is mediated mainly through activation of AMPK and inhibition of transcription factor activator protein 1 (AP1) and NF-κB [84]. For example, berberine inhibits mucosal generation of interleukin-8 (IL-8), which is responsible for polymorphonuclear neutrophils infiltration in intestinal lesions of intestine bowel disease (IBD) and ulcerative colitis [85]. Similar effects were observed in metformin. Study of Koh et al indicates that metformin inhibits NF-κB activation in intestinal epithelial cells and ameliorates colitis-related carcinogenesis [86]. IBD, colitis and the Crohn’s disease are characterized by chronic inflammation caused by infection or autoimmune dysregulation, which result in increased risk of cancer [87]. Furthermore, macrophage infiltration into tumour tissues has been depicted to have a role in tumorigenesis and tumour progression [88]. In light of the anti-tumour activities mentioned above, simultaneous treatment of inflammation and cancer by berberine or metformin may yield a better prognosis and point to a new research area [89].

Obesity-associated insulin resistance and β cell dysfunction can induce serious inflammation [90]. Berberine via activation of AMPK can exert an anti-inflammatory effect in adipose tissue induced by high fat diet. The obesity is accompanied by a low-grade chronic inflammation state, characterized by infiltration of CD11c+ macrophages or adipose tissue macrophages (ATM) and neutrophils [91–93]. IL-1β and IL-18 as principal inflammatory cytokines released from ATM contribute to the obesity-associated insulin resistance. Berberine improves insulin sensitivity by upregulating autophagic levels in macrophage and inhibiting ATM phenotypic switch to decrease CD11c+ population [94, 95]. Likewise, metformin is able to decrease production of nitric oxide, prostaglandin E2 and proinflammatory cytokines (IL-1β, IL-6 and tumour necrosis factor (TNF)-α) through inhibition of NF-κB activation in macrophages [96]. The anti-inflammation effects of berberine and metformin can inhibit the progress of diseases, which gives better outcomes for patients [97].

## MECHANISMS UNDERLYING THE ACTIONS OF METFORMIN AND BERBERINE

Metformin was first reported to implement its function through activation of AMPK [98]. This occurs via inhibition of complex I in the mitochondrial respiratory chain, leading to an increase in the AMP/ATP ratio and thereby allowing AMP to bind and activate AMPK [99]. In some cases, however, this notion was challenged by two observations. First, hepatic ablation of LKB1 does not abolish the inhibitory effect of metformin on hepatic gluconeogenesis [100]. Second, *in vitro* studies revealed that the concentration of metformin required for activation of AMPK is greater than that for acute inhibition of gluconeogenesis in hepatocytes [98]. These findings have led to scrutiny of the mechanism underlying inhibition of hepatic gluconeogenesis. Thus, Madiraju et al have demonstrated that metformin inhibits mitochondrial glycerol-3-phosphate dehydrogenase, leading to changes in mitochondrial and cytoplasmic redox states and glucose synthesis [23]. This takes place independently of AMPK activation. In addition, other mechanisms such as AMP-medicated inhibition of adenylate cyclase have been delineated, a mechanism by which metformin inhibits glucagon-induced glucose production in liver, an AMPK-independent action [101]. Which mechanism prevails probably depends on the context and function of specific tissues or cells. Since gluconeogenesis is insignificant in other cells than hepatocytes, it is conceivable many of other actions of metformin is still mediated by AMPK in these cells.

Berberine has been shown to inhibit mitochondrial respiratory complex I, which could lead to the increase of AMP and subsequent AMPK activation [102]. However, a recent report demonstrates that berberine blocks complex I, leading to increases in glucose consumption and lactate release, which is independent of AMPK [103]. This raises an interesting possibility that berberine and metformin may act through similar mechanisms despite different structure and transporters. As inhibition of respiratory chain engenders stress conditions, addition of berberine or metformin could elicit cellular stress responses, such as activation of p38 and JNK pathways, which might be dependent or independent of AMPK activation. Also, we cannot exclude extra-mitochondrial events evoked by these drugs. It is not clear whether metformin and berberine undertake all actions via the same mechanisms, or some via similar and others different mechanisms.

## PHARMACOKINETICS

Metformin is an excellent drug with good oral bioavailability and pharmacokinetic behaviours. It is usually not metabolized in our body and its half-life is about 5 hours. Fixed forms of drugs are excreted in urine with a renal clearance of 510 ± 120 ml/min [104]. Following absorption, metformin is rapidly distributed in various tissues without binding to plasma proteins, and then undergoes hepatic uptake and renal excretion, which are mainly controlled by organic cation transporters (OCTs) [105, 106]. These transporters manage the influx and efflux of metformin in different tissue cells to maintain steady state concentrations of metformin. OCT1 (*SLC22A1*) and OCT3 (*SLC22A3*) are expressed on the basolateral membranes of hepatocytes [107–109] and in skeletal muscle [110–112]. The clearance of metformin is finished by kidney. Metformin is transported into the proximal tubular lining cells by OCT1, OCT2 and OCT3 on basolateral side of renal tubular cells [105]. Then the H (+)/organic cation antiporters, multidrug and toxin extrusion 1(MATE1) and MATE2K are responsible for the transport of metformin from the renal cells to the urine in tubule lumen [113]. Thus, it should be cautious to use metformin in patients with impaired liver or kidney function. Recently, it has been demonstrated that genetic polymorphisms in transporter genes play an important role in both metformin pharmacokinetics and pharmacodynamics [114, 115].

Of note, the steady state concentrations of metformin in plasma are very low. In an adult T2DM patient by oral administration of 1.5∼2.5 g/day (∼30 mg/kg/day), plasma concentration is approximately 10 mM in plasma [105]. In rodent animals, usually 5 times injection dose (150 mg/kg, i.p.) or 10 times oral dose (250∼300 mg/kg) of humans will achieve 5∼10 mM which generate similar effects [116]. These low concentrations can hardly lead to acute activation of AMPK although gluconeogenesis is inhibited. One explanation is that metformin is a cation and transport to mitochondria slowly, but accumulates there to high concentrations (100∼500 fold, i.e. 1∼5 mM), which is sufficient for AMPK activation [116]. In keeping with this, the concentrations required for AMPK activation *in vitro* are at mM levels.

Concentrations of berberine that are effective in *in vitro* studies range between 10∼100 mM, which are thousands of times greater than those normally attainable following oral ingestion in human [117]. Berberine has poor solubility in aqueous solution and its solubility at 37^°^C depends on pH, which decreases with lowering pH and reaches maximum of 9.69 ± 0.37 mM in phosphate buffer [118, 119]. It is absorbed from gastrointestinal tract with low efficiency. The maximum concentration (Cm) of berberine in plasma is 12 nM after the oral administration of 100 mg/kg in rats [120]. The Cm is 50 nM at 15 min after the oral administration of 25 mg/kg in rats and the plasma concentration decreases rapidly within 12 h [121]. In humans, a single oral dose of 500 mg berberine administration generates 0.07±0.01 nM in plasma, while the Cm is 4.0±2.0 nM after chronic administration of 15 mg/kg for three months [122]. Such low concentrations attained *in vivo* raise a question as to if they could take effect. It is possible that berberine might accumulate inside cells, similarly to metformin, to achieve the *in vivo* effects.

The low efficiency of berberine transport to blood is mostly accounted for by the poor oral bioavailability, which is determined by several factors [123]. First, drug self-aggregation decreases the solubility. Berberine tends to aggregate, especially in low pH, which leads to poor absorption in stomach and upper small intestine [119]. Second, the permeability of berberine is low [118]. Third, berberine is a substrate of P-glycoprotein-mediated efflux in the intestine, which further limits its transport in the absorption direction [124]. 80% of berberine is metabolized in the liver and intestine by CYP2D6. These together define the difficulty in delivery of berberine. Therefore, to enhance the efficacy, it is imperative to enhance the bioavailability by modifying structure of the drug or enhancing permeation with additives.

## PROSPECTIVE

Hyperglycaemia, hyperlipidemia, insulin resistance and obesity are collectively defined as metabolic syndrome (MetS), firstly termed by Haller and Hanefeld in 1975 [125]. These metabolic abnormalities are strongly interrelated, ameliorating one could benefit the others. Otherwise the culminating adverse outcome significantly increases risk of T2DM, CVD, and tumours [126]. Metformin has been used in clinics for years and its impacts on various metabolic disorders as mentioned above make it stand out in MetS therapies. In recent years, berberine is emerging as an attractive new agent in treating MetS, as it shares many features with metformin and more importantly, is also low cost. However, the poor oral bioavailability may limit its application [27]. Application of metformin in optimal dosage may also be prevented in OCT1 variant patients where adverse gastrointestinal reactions are observed [127]. In the case that is refractory or intolerable to metformin, berberine may be used as an alternative or additive agent to increase tolerance and minimize the side effects. In keeping with this, a recent study on a six months trial with 60 patients with T2DM randomly divided groups of berberine and metformin or metformin alone and better efficacy was observed in the combined treatment group [25]. It is postulated that the synergism action of berberine and metformin is attributed to similar anti-diabetic mechanisms in spite of different transporter and metabolism. Therefore, combination of these two drugs might allow reduction in dosage of each individual drugs to solve problems such as oral bioavailability of berberine and side effects of each alone.

## CONCLUSIONS

Metformin and berberine shares many aspects in actions and mechanisms despite different structure. While metformin is the first line anti-diabetic drug, berberine is not extensively used in treatment of T2DM and MetS in Western countries although it is a nonprescriptive medicine and experimental studies show promising results. Few studies directly compare the efficacy of metformin and berberine in treating T2DM and MetS. Since the side effects of berberine are tolerable and controllable (usually gastrointestinal discomfort), clinical investigations to compare these two drugs are definitely feasible. Hopefully, more clinical investigation on the use of berberine will be conducted in the near future.

## Abbreviations

AMPK: AMP-activated protein kinase
ATM: adipose tissue macrophages
EMT: epithelial and mesenchymal transition
HbA1c: hemoglobin A1c
IL-1b: interleukin-1b
IL-6: interleukin-6
LDLR: low density lipoprotein receptor
LKB1: liver kinase B1
MAPK: mitogen-activated protein kinase
MATE: multidrug and toxin extrusion
MetS: metabolic syndrome
mTOR: mammalian target of rapamycin
NAFLD: non-alcoholic fat liver disease
NASH: non-alcoholic steatohepatitis
NF-κB: nuclear factor-kappa B
OCT: organic cation transporter
PCSK9: proprotein convertase subtilisin/kexin type 9
PKC: protein kinase
PPARγ: peroxisome proliferator-activated receptor gamma
RARβ: retinoic acid receptor beta
T2DM: type 2 diabetes mellitus
TSC1: tuberous sclerosis complex 1
TSC2: tuberous sclerosis complex
UKPDS: the United Kingdom Prospective Diabetes Study
